# Preliminary investigation of the effect of non-cardiac surgery on intraoperative islet and renal function: a single-center prospective cohort study

**DOI:** 10.3389/fmed.2024.1235335

**Published:** 2024-02-13

**Authors:** Yongtao Sun, Xiaoning Zhang, Min Zhang, Yongle Guo, Tao Sun, Mengjie Liu, Xiaojun Gao, Yang Liu, Zhongquan Gao, Lina Chen, Xiaoyan Du, Yuelan Wang

**Affiliations:** ^1^Department of Anesthesiology, The First Affiliated Hospital of Shandong First Medical University and Shandong Provincial Qianfoshan Hospital, Shandong Institute of Anesthesia and Respiratory Critical Medicine, Jinan, China; ^2^Shandong First Medical University and Shandong Academy of Medical Sciences, Jinan, China; ^3^Department of Clinical Laboratory Medicine, The First Affiliated Hospital of Shandong First Medical University and Shandong Provincial Qianfoshan Hospital, Jinan, China; ^4^Yidu Cloud (Beijing) Technology Co. Ltd., Beijing, China; ^5^Department of Anesthesiology, Shandong Provincial Hospital Affiliated to Shandong First Medical University (Shandong Provincial Hospital), Jinan, China

**Keywords:** non-cardiac surgery, intraoperative, different operation methods, islet function, renal function

## Abstract

**Background:**

The effect of different non-cardiac surgical methods on islet and renal function remains unclear. We conducted a preliminary investigation to determine whether different surgical methods affect islet function or cause further damage to renal function.

**Methods:**

In this prospective cohort study, the clinical data of 63 adult patients who underwent non-cardiac surgery under general anesthesia were evaluated from February 2019 to January 2020. Patients were divided into the abdominal surgery group, the laparoscopic surgery group, and the breast cancer surgery group. The primary outcome was the difference between the effects of different surgical methods on renal function.

**Results:**

Islet and renal function were not significantly different between the groups. The correlation analysis showed that hematocrit (HCT) and hemoglobin (HB) were negatively correlated with fasting plasma glucose (FPG) (*p* < 0.05), MAP was positively correlated with C-peptide (*p* < 0.05), and HCT and Hb were positively correlated with serum creatinine (SCr) (*p* < 0.05). Fasting insulin (FINS) and C-peptide were negatively correlated with SCr (*p* < 0.05), and the homeostatic model assessment of insulin resistance (HOMA-IR) was positively correlated with SCr (*p* < 0.05). FINS, C-peptide, HOMA-IR, and the homeostatic model assessment of β-cell function (HOMA-β) were positively correlated with cystatin C (Cys C) (*p* < 0.05).

**Conclusion:**

FINS, C-peptide, and HOMA-IR had positive effects on beta-2-microglobulin (β_2_-MG). FINS, C-peptide, and HOMA-IR were positively correlated with Cys C and β_2_-Mg. While FINS and C-peptide were negatively correlated with SCr, HOMA-IR was positively correlated with SCr.

## 1 Introduction

Surgeries stimulate visceral sympathetic nerves, which can increase the synthesis and release of adrenal glucocorticoids through the hypothalamic–pituitary axis, inhibit the utilization of glucose in tissues, antagonize the effect of insulin, and cause stress, insulin resistance (IR), and hyperglycemia, placing the nervous system, endocrine system, and immune system in a state of stress. As a result, the patient’s resistance ability is reduced, and the generation of peroxide and oxygen-free radicals is increased, thereby increasing the risks of postoperative complications and surgery (1, 2). Serum insulin and C-peptide levels may reflect the secretory function of islet β cells. Mechanical stimulation of surgeries and the high-pressure environment of laparoscopic surgeries may stimulate islet β cells and inhibit their secretion. Acute disease, injury, or surgical tissue trauma will lead to changes in glucose metabolism and induce a neuroendocrine stress response, resulting in insulin resistance, increased gluconeogenesis, glycogenolysis, and decreased peripheral glucose uptake (3, 4). The renin-angiotensin system (RAS) involves many physiological and pathophysiological processes. Increasing evidence has supported the involvement of the RAS in expression in human and rodent pancreases (5). In addition, angiotensinogen is the substrate of renin, which is mainly expressed in α cells (which mainly secrete glucagon) (6).

The prevalence of chronic kidney disease (CKD) has increased worldwide, exceeding 10% (7), and it is even higher among patients who undergo surgery. Acute kidney injury (AKI) remains a common perioperative complication that affects the short- and long-term outcomes of patients. Strategies to prevent AKI in high-risk patients include avoiding nephrotoxic drugs and contrast agents, managing hyperglycemia, and closely monitoring renal function (8). Both hyperglycemia and insulin resistance can affect renal function. Diabetic nephropathy (DN) is the most common microvascular complication of diabetes and is the main cause of high mortality and disability in DM patients (9). At present, there are very few relevant studies investigating the effect of surgical methods on islet and renal function. For this reason, we hypothesized that the stress response secondary to surgical anesthesia induces hyperglycemia, which affects islet cell sensitivity and leads to insulin resistance, ultimately resulting in kidney damage.

## 2 Methods

This was a single-center prospective cohort study. The study was conducted in accordance with the Declaration of Helsinki (World Medical Association 2013) and was approved by the local Ethics Committee of Qianfoshan Hospital, Shandong Province (YXLL-KY-2019 (036)). The study was nationally registered (Chinese Clinical Trials.gov: ChiCTR 1,900,028,686, 30 December 2019). All patients signed informed consent forms prior to enrollment in the study.

### 2.1 Patients

Patients undergoing non-cardiac surgery, including abdominal surgery or radical mastectomy for breast cancer, in the First Affiliated Hospital of Shandong First Medical University from February 2019 to January 2020 were selected. Abdominal surgery included open surgery and laparoscopic surgery. According to the surgical method, patients were assigned to the abdominal surgery group, the laparoscopic surgery group, or the breast cancer surgery group.

The baseline HR, NBP, SpO_2_, and bifrequency index (BIS) values (BIS^®^ Monitor, Covidien, Boulder, CO, USA) were routinely monitored and recorded during the patients’ operations. Midazolam 0.05 mg kg^−1^, etomidate 0.2 mg kg^−1^, sufentanil 0.4 μg kg^−1^, and rocuronium 0.6 mg kg^−1^ were intravenously injected in sequence. TCI propofol 1.5–3.5 μg ml^−1^ combined with sevoflurane using a minimal alveolar concentration (MAC) of 1.0 and remifentanil pumped at a constant rate of 0.1–1 μg kg^−1^ min^−1^ were applied to maintain anesthesia, and rocuronium was used intermittently. EtCO_2_ was kept between 35 and 45 mmHg, and BIS values were kept between 40 and 60 during surgery. Intraoperative fluids contained no dextrose. Any case of intraoperative hypotension (MAP <65 mmHg) or bradycardia (HR < 50 beats/min) was treated with an intravenous injection of ephedrine 5 mg or atropine 0.4 mg.

### 2.2 Inclusion criteria

Patients who were between 18 and 70 years of age with an ASA classification of I–II and who underwent level 4 abdominal surgery (including radical gastrectomy, radical resection of rectal cancer, resection of liver tumor, hysterectomy, etc.) or breast cancer surgery were included in the study.

### 2.3 Exclusion criteria

Patients who had a history of diabetes or had taken hypoglycemic drugs 3 months before surgery; patients with a history of circulatory diseases such as hypertension or heart disease; patients with a history of liver dysfunction or liver surgery; patients with a history of drug allergy; patients who suffered from shock, acidosis, or any other crises during the operation; patients who were treated with blood products or vasoactive drugs for rescue; patients who voluntarily withdrew from the study; patients with water and electrolyte disorders or an acid–base imbalance; and patients who received immunotherapy.

### 2.4 Primary outcome

The primary outcome was renal function injury. SCr, Cys C, and β_2_-MG were used to assess renal function. The observation time points were preoperation (baseline), after the induction of anesthesia, and 30 and 60 min after the surgery. Five milliliters of venous blood were collected.

### 2.5 Secondary outcomes

The secondary outcomes included islet function (FPG, FINS, C-peptide, and HOMA-IR, HOMA-β, and HOMA of insulin sensitivity [HOMA-IS]); correlation analysis of intraoperative islet function and renal function; and Hb, HCT, and MAP were obtained from arterial blood gas before surgery, after induction of anesthesia, and 30 min and 60 min after surgery. The incidence of outliers for each indicator induction of anesthesia 30 min and 60 min after surgery (occurrence ≥1, recorded as outliers). HOMA-IR, HOMA-β, and HOMA-IS were used to assess islet function, which was calculated by a homeostasis assessment model with FPG, FINS, and C-peptide, such as HOMA-IR = (FINS × FPG)/22.5, HOMA-β = 20 × FINS/(FPG-3.5), and HOMA-IS = 1/(FPG × FINS).

### 2.6 Sample size

The sample size was calculated using data from the Long TE study (10), an observational case-control study examining the incidence, risk factors, and mortality in patients with AKI following abdominal surgery, including exploratory laparotomy (12.9%), laparoscopic surgery (3.4%), and minor surgical procedures (4.1%), the sample size was calculated. A sample size of 344 achieves 80% power to detect an effect size (W) of 0.1675 using two degrees of freedom chi-square test with a significance level (alpha) of 0.05 by sample size by PASS 15.0. The study of Long TE observed that the incidence of AKI in all abdominal procedures was 6.8%. Therefore, we assumed that intraoperative AKI was 1/6 of postoperative AKI, and we estimated that the sample size should be 57 cases. With a 10% shedding rate, we needed to include at least 63 subjects (at least 21 in each group).

### 2.7 Statistical analysis

A comparison of the preoperative baseline data was performed. For continuous variables, the differences between the groups were compared using a one-way ANOVA or the Kruskal–Wallis non-parametric test according to whether the data met a normal distribution. A chi-square test was used to analyze the differences between the groups for categorical variables.

To compare the differences between three groups of patients with the same intraoperative index at the same time, a one-way ANOVA, or the Kruskal–Wallis non-parametric test, was conducted according to whether the data met the normal distribution, and multiple comparisons between the groups were conducted.

Repeated-measures ANOVA was used to compare the changes in the same index at different times in patients within the same group. The trend of the same index over time in the three groups during surgery was compared using a multivariate analysis of variance and Pillai’s trace test, and the variation trend is shown as a broken line graph.

To explore the effects of the different surgical methods on islet and renal function in patients under general anesthesia and to analyze the correlation between islet and renal function, the MIXED procedure of SAS was used to perform a parameter estimation of the MIXED linear model with time-dependent covariate repeated measurement data.

SAS (SAS Institute, Inc., Cary, NC, SAS 9.4 Version) was used for the statistical analysis. Unless otherwise specified, α was 0.05 (two-sided test), and *p* < 0.05 was defined as statistically significant.

## 3 Results

### 3.1 Trial cohort and patient flow

**Figure 1:**
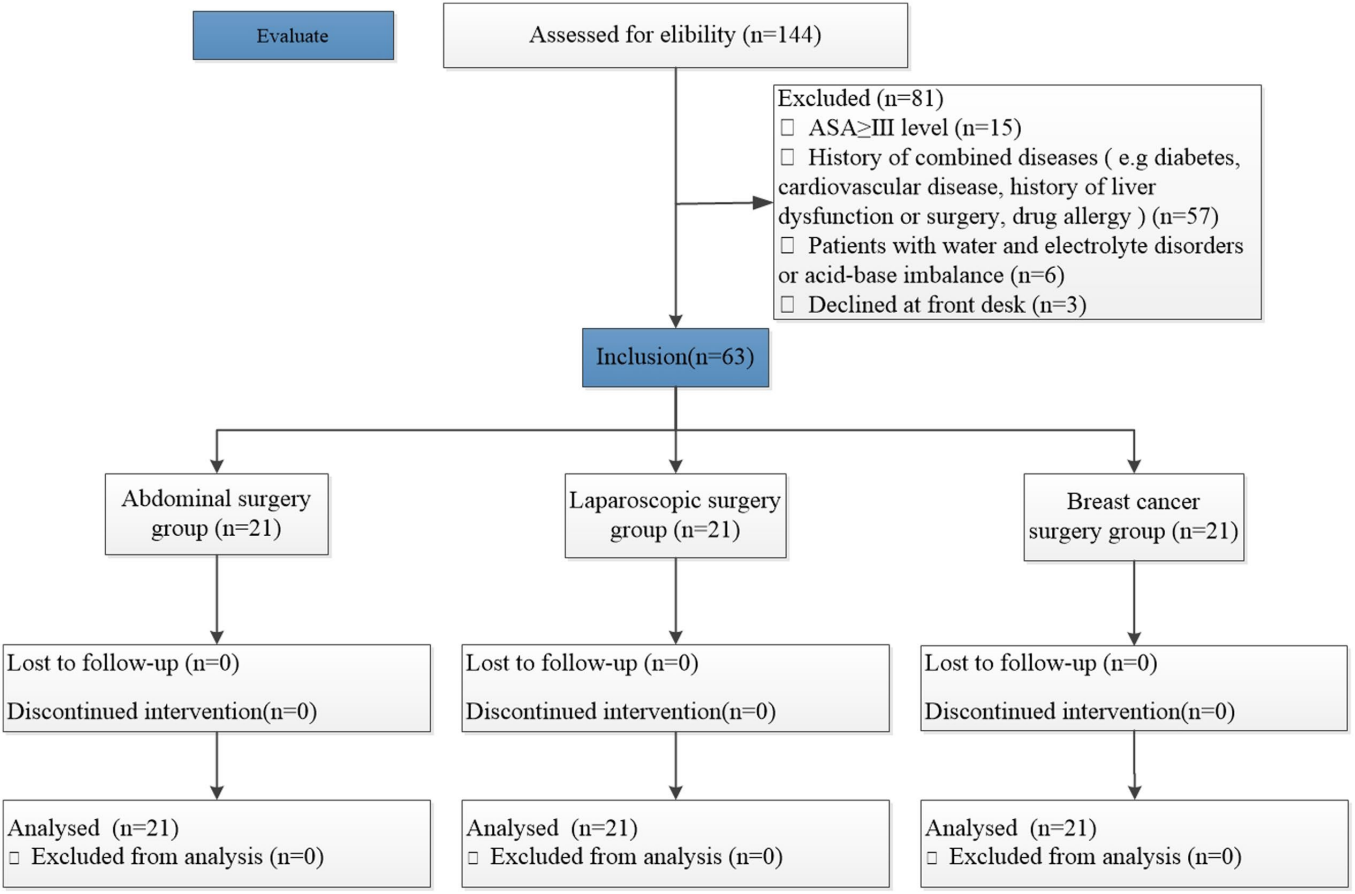
CONSORT flow diagram.

The inclusion and exclusion criteria for the patients are shown in Figure 1. Between February 2019 and January 2020, a total of 144 patients aged 18–70 who underwent abdominal or breast surgery were initially recruited after assessment of the eligibility criteria. However, 81 patients were excluded (the abdominal surgery group included 34 patients, the laparoscopic surgery group included 31 patients, and the breast cancer surgery group included 16 patients); 63 patients were finally included in the study.

### 3.2 Baseline characteristics

There were no statistically significant differences in age, weight, body mass index (BMI), or globulin (GLB) among the three groups, while there were statistically significant differences in total protein (TP) and albumin (ALB) between the breast group and the open surgery group (*p* < 0.05). This study compared the breast cancer group with the abdominal surgery group to investigate the differences in islet and renal function between surface surgeries and abdominal surgeries. However, because all the breast cancer patients were female, there was a significant difference between the other two groups of patients. Since islet and renal function are not affected by sex, this difference is not discussed in this study (Table 1).

**Table 1:** Baseline characteristics.

	Abdominal surgery group (*n* = 21)	Laparoscopic surgery group (*n* = 21)	Breast cancer surgery group (*n* = 21)	*p*-value
Age (years)	62 (50, 64)	49 (45, 53)	54 (44, 60)	0.1453^a^
Sex				<0.0001^b^
Male	14 (67%)	9 (43%)	0 (0%)	
Female	7 (33%)	12 (57%)	21 (100%)	
Weight (kg)	65 (58.0, 71.5)	64 (60.0, 75.0)	62.75 (59.0, 69.5)	0.8572
BMI (kg/m^2^)	23.2 (21.6, 25.0)	24.2 (21.6, 26.9)	24.5 (23.2, 27.2)	0.1495
TP (g/L)	66.7 (63.8, 72.9) *	71.8 (65.4, 76.0)	76 (69.8, 77.8) *	0.0078
ALB (g/L)	42.9 (39.7, 44.5) *	45.5 (41.5, 46.6)	47.7 (43.4, 49.6) *	0.0129
GLB (g/L)	24.9 (23.1, 26.1)	27.2 (23.8, 28.6)	27.8 (26.1, 30.4)	0.0875

Data are expressed as the median (Q1, Q3) or *n* (%). BMI, body mass index; TP, total protein; ALB: albumin; GLB, globulin. The significance was set at *p* < 0.05. The symbol * indicates that the difference between the two groups was statistically significant in the multiple comparative analysis.

^a^One-way ANOVA or Kruskal–Wallis *H* test.

^b^Chi-square test.

### 3.3 Primary outcome

There was no significant difference in β_2_-MG or Cys C among the three groups at the same time point. At 60 min after surgery, the SCr of the abdominal surgery group and the laparoscopic surgery group was significantly higher than that of the breast cancer surgery group [69 (52, 81) vs. 51 (45, 58), 61 (53, 71) vs. 51 (45, 58), *p* = 0.0093], and there was no significant difference between the abdominal surgery group and the laparoscopic surgery group (Table 2).

**Table 2:** Renal function indexes.

	Abdominal surgery group (*n* = 21)	Laparoscopic surgery group (*n* = 21)	Breast cancer surgery group (*n* = 21)	*P*-value
SCr (μmol/L)				
Preoperative	68 (56, 78)	61 (54, 76)	57 (48, 63)	0.0635
After anesthesia induction	64 (48, 72) *	52 (49, 68) *	51 (48, 59) *	0.1142
30 min after surgery	63 (50, 76) *	56 (50, 68) *	52 (47, 59) *	0.0933
60 min after surgery	69 (52, 81) ^#^	61 (53, 71)	51 (45, 58) *^#†^	0.0093
Cys C (mg/L)				
Preoperative	0.75 (0.67, 0.88)	0.73 (0.62, 0.78)	0.72 (0.61, 0.80)	0.3482
After anesthesia induction	0.73 (0.62, 0.82) *	0.69 (0.62, 0.77)	0.70 (0.57, 0.73) *	0.5508
30 min after surgery	0.72 (0.63, 0.81) *	0.74 (0.63, 0.77)	0.71 (0.59, 0.75)	0.7475
60 min after surgery	0.73 (0.61, 0.83)	0.71 (0.62, 0.79)	0.71 (0.58, 0.74)	0.5872
β_2_-MG (mg/L)				
Preoperative	1.96 (1.56, 2.17)	1.63 (1.36, 1.95)	1.63 (1.39, 1.92)	0.1701
After anesthesia induction	1.81 (1.42, 2.00) *	1.56 (1.34, 1.80)	1.50 (1.33, 1.84) *	0.22
30 min after surgery	1.79 (1.52, 2.06)	1.60 (1.34, 1.83)	1.49 (1.32, 1.93)	0.2329
60 min after surgery	1.66 (1.33, 1.92) *	1.58 (1.40, 1.90)	1.44 (1.31, 1.80) *	0.4505

*p*-values were derived from ANOVA or the Kruskal–Wallis test for continuous variables a. The significance was set at *P* < 0.05. SCr: serum creatinine; Cys C, cystatin C; β_2_-MG, beta-2-microglobulin. *p < 0.05 indicates a statistically significant difference between different times in the same group; ^#^*p* < 0.05 indicates the difference between the two groups was statistically significant in the multiple comparative analysis; ^†^*p* < 0.05 indicates the difference between this group and the other two groups was statistically significant.

**Figure 2:**
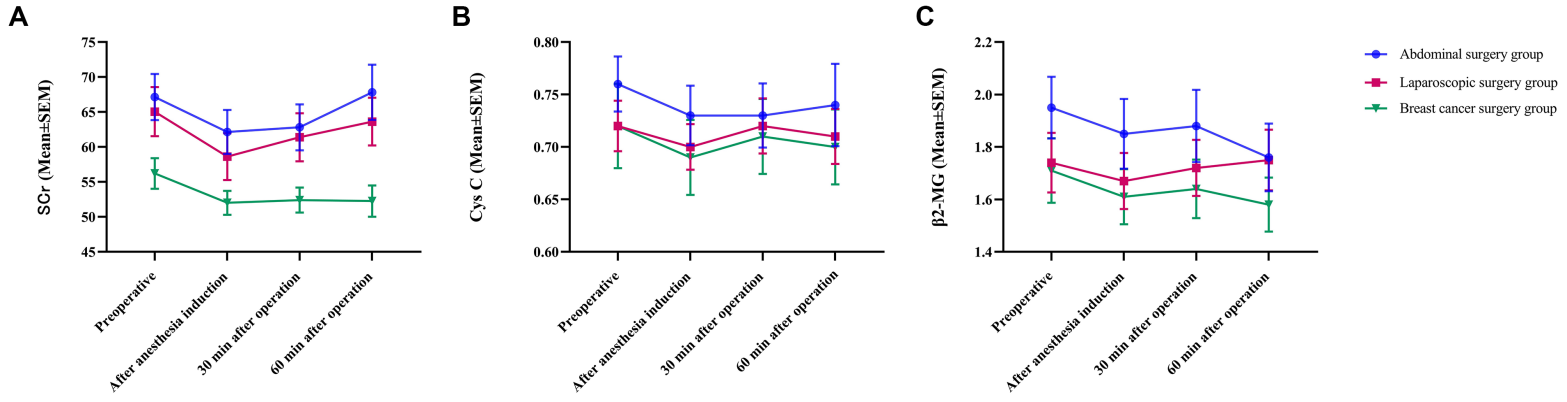
Trend chart of islet function. All data are presented as the mean ± SEM.

The SCr, Cys C, and β_2_-MG levels decreased after anesthesia induction. Thirty minutes after surgery, SCr, Cys C, and β_2_-MG increased to varying degrees and did not exceed their respective values before surgery (Figure 2). Sixty minutes after surgery, the levels of β_2_-MG in the laparoscopic surgery group continued to increase and exceeded the preoperative level, while the levels of β_2_-MG in the abdominal surgery group and the breast cancer group continued to decrease and were lower than the preoperative level (Figure 2A). The SCr levels of the patients in the abdominal surgery group and the patients in the laparoscopic surgery group continued to increase and were similar to the preoperative level, while the level of SCr in the patients in the breast cancer group continued to decrease and was lower than the preoperative level (Figure 2B). The level of Cys C in the patients in the abdominal surgery group continued to increase, remaining at the same level as before surgery, while the levels of Cys C in patients in the laparoscopic surgery group and the breast cancer group continued to decrease and were lower than the preoperative level (Figure 2C). There was no significant difference in the results of the different groups at the different time points, and the trends of the three groups were basically the same.

### 3.4 Secondary outcomes

There was no significant difference in FPG, FINS, C-peptide, HOMA-IR, or HOMA-IS among the three groups at the same time point. At 30 and 60 min after surgery, the HOMA-β of the laparoscopic surgery group was significantly higher than that of the abdominal surgery group [36.71 (21.88, 58.17) vs. 66.00 (50.00, 83.04), *p* = 0.0103; 47.53 (31.19, 89.50) vs. 93.47 (55.14, 123.79), *p* = 0.0210] (Table 3).

**Table 3:** Islet function indexes.

	Abdominal surgery group (*n* = 21)	Laparoscopic surgery group (*n* = 21)	Breast cancer surgery group (*n* = 21)	*p*-value
FPG (mmol/L)				
Preoperative	5.6 (5.2, 5.8)	5.2 (4.5, 5.6)	5.7 (5.2, 5.8)	0.0637
After anesthesia induction	5.3 (4.8, 5.5) *	4.9 (4.3, 5.4) *	5.3 (5.1 t, 5.7) *	0.2558
30 min after surgery	5.7 (5.2, 6.1) *	5.2 (4.5, 6.0)	5.4 (5.1, 5.7)	0.1317
60 min after surgery	5.7 (5.3, 6.2) *	5.4 (4.4, 6.3) *	5.4 (5.1, 5.8)	0.2931
FINS (μIU/ml)				
Preoperative	5.88 (2.88, 8.26)	5.66 (3.77, 7.78)	6.28 (4.84, 8.10)	0.8734
After anesthesia induction	3.12 (1.59, 4.83) *	3.71 (2.52, 5.05) *	5.34 (3.32, 7.21)	0.0633
30 min after surgery	4.99 (3.73, 10.52)	5.65 (3.74, 11.94) *	4.94 (4.41, 9.30)	0.7825
60 min after surgery	7.05 (4.44, 11.76) *	5.21 (3.42, 10.13)	5.62 (4.35, 11.37)	0.6421
C-peptide (ng/ml)				
Preoperative	1.77 (1.16, 1.92)	1.58 (1.41, 2.06)	1.45 (1.12, 1.89)	0.4069
After anesthesia induction	1.30 (1.01, 1.71) *	1.43 (1.15, 1.64) *	1.59 (1.07, 1.93)	0.7238
30 min after surgery	1.44 (1.11, 2.17)	1.33 (1.14, 2.12)	1.72 (1.47, 2.31) *	0.4606
60 min after surgery	1.9 (1.30, 2.68) *	1.35 (1.09, 2.66)	1.76 (1.40, 2.63) *	0.3055
HOMA-IR				
Preoperative	1.36 (0.72–2.08)	1.31 (0.78–2.09)	1.46 (1.21–2.05)	0.8187
After anesthesia induction	0.72 (0.38–1.22) *	0.71 (0.46–1.20) *	1.34 (0.75–1.63)	0.0695
30 min after surgery	1.44 (0.87–2.71) *	1.33 (0.86–3.24)	1.29 (0.96–2.21)	0.9852
60 min after surgery	1.82 (1.18–3.24) *	1.25 (0.75–2.84) *	1.32 (1.03–2.83)	0.5942
HOMA-β				
Preoperative	56.00 (4.20, 80.50)	75.40 (47.15, 104.00)	69.26 (39.69, 75.09)	0.2235
After anesthesia induction	36.71 (21.88, 58.17) ^#^	66.00 (50.00, 83.04) *^#^	54.34 (40.27, 83.98)	0.0103
30 min after surgery	47.53 (31.19, 89.50) *^#^	93.47 (55.14, 123.79) ^#^	65.21 (48.67, 94.53)	0.0210
60 min after surgery	70.05 (36.90, 88.13)	56.00 (44.00, 102.25)	67.57 (47.45, 102.16) *	0.6770
HOMA-IS				
Preoperative	0.03 (0.02, 0.06)	0.03 (0.02, 0.06)	0.03 (0.02, 0.04)	0.8187
After anesthesia induction	0.06 (0.04, 0.12) *	0.06 (0.04, 0.10)	0.03 (0.03, 0.06)	0.0695
30 min after surgery	0.03 (0.02, 0.05)	0.03 (0.01, 0.05)	0.03 (0.02, 0.05)	0.9852
60 min after surgery	0.02 (0.01, 0.04) *	0.04 (0.02, 0.06)	0.03 (0.02, 0.04)	0.5942

*P*-values were derived from ANOVA or the Kruskal–Wallis test for continuous variables a. The significance was set at *P* < 0.05. FPG: fasting plasma glucose; FINS: fasting insulin; HOMA-IR: homeostatic model assessment of insulin resistance; HOMA-β: homeostatic model assessment of β-cell function; HOMA-IS: homeostatic model assessment of insulin sensitivity. *p < 0.05 indicates a statistically significant difference between different times in the same group; ^#^*p* < 0.05 indicates the difference between the two groups was statistically significant in the multiple comparative analysis.

**Figure 3:**
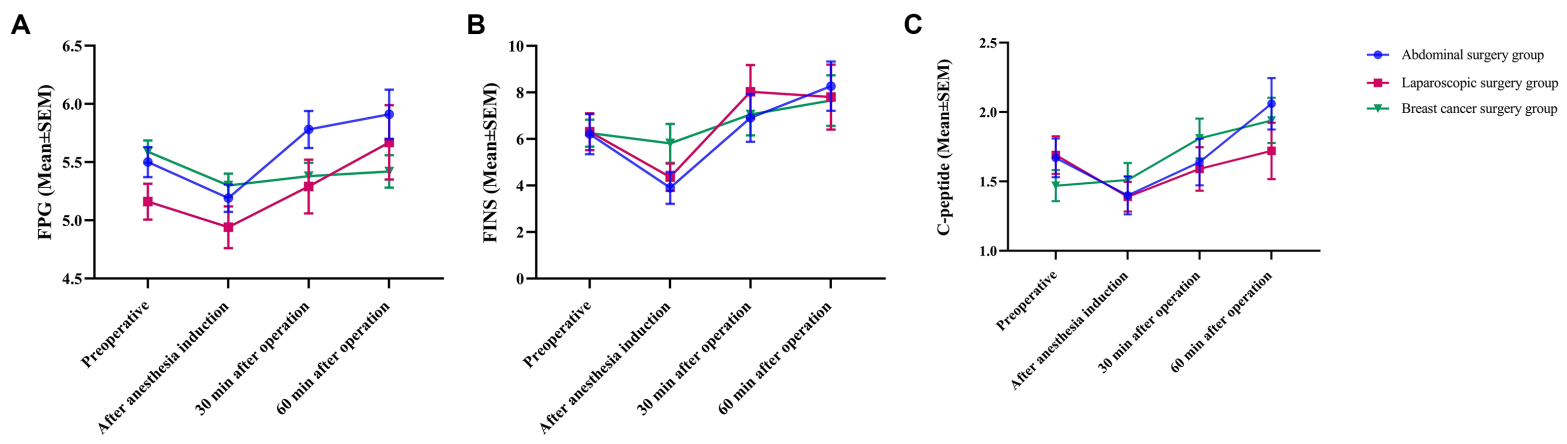
Trend chart of renal function. All data are presented as the mean ± SEM.

After anesthesia induction, FPG decreased initially and then gradually increased 30 min after surgery. The FPG in the abdominal surgery group and laparoscopic surgery group reached the highest point at 60 min after surgery and was higher than the FPG preoperatively. A decreasing trend during surgery was observed in the breast cancer group (Figure 3A). After anesthesia induction, the FINS decreased first, while the FINS in the breast cancer group decreased slightly, gradually increased 30 min after the surgery, reached the highest point at 60 min after the surgery, and were higher than the FINS at the time before the operation (Figure 3B). In the abdominal surgery group and laparoscopic surgery group, C-peptide showed a downward trend after anesthetic induction and gradually increased 30 min after surgery. Sixty minutes after surgery, the C-peptide level in the abdominal surgery group increased to the highest point, which was higher than the C-peptide level at the time before surgery. In the laparoscopic surgery group, it was lower than the C-peptide level at the time before surgery. The C-peptide levels in the breast cancer group continued to increase during surgery (Figure 3C). There were no significant differences among the different groups at the different time points, and the trends of the three groups were basically the same.

**Figure 4:**
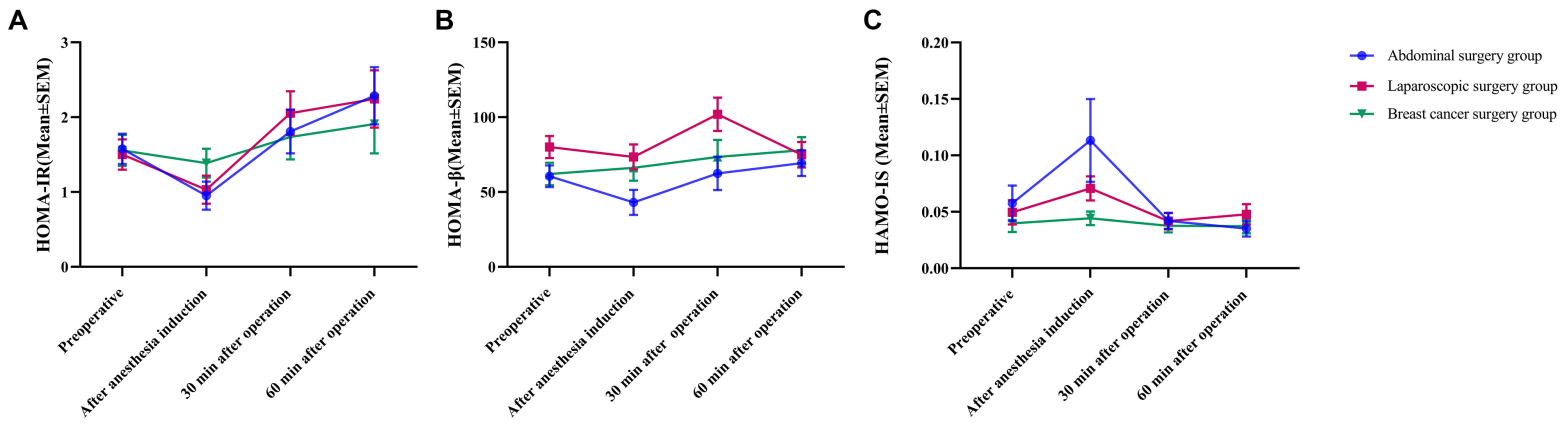
Trend chart of islet function. All data are presented as the mean ± SEM.

HOMA-IR initially decreased after the induction of anesthesia, gradually increased 30 min after surgery, reached the highest point at 60 min after surgery, and was higher than HOMA-IR before the operation. HOMA-β initially decreased after anesthetic induction in the abdominal surgery group and laparoscopic surgery group and increased gradually 30 min after surgery (Figure 4A). At 60 min after surgery, HOMA-β continued to increase in the abdominal surgery group, while in the laparoscopic surgery group, it decreased and was lower than the preoperative level. In the breast cancer group, HOMA-β continuously increased. HOMA-IS increased to the maximum value after anesthetic induction and decreased to the minimum value at 30 and 60 min after surgery in both the abdominal surgery group and breast cancer group (Figure 4B). In the laparoscopic surgery group, the value decreased to its lowest at 30 min after surgery and gradually increased 60 min after surgery. There was no significant difference in the results of the different groups at the different time points, and the trend of the three groups was basically the same (Figure 4C).

Compared with the preoperative values, MAP, HCT, and Hb decreased to different degrees after anesthetic induction and at 30 and 60 min after surgery, and the differences were statistically significant (*p* < 0.05). At 60 min after surgery, the HCT of the abdominal surgery group was significantly higher than that of the breast cancer surgery group. The differences were statistically significant [36 (34, 38) vs. 32 (30, 36), *p* < 0.05], except for the differences in MAP, HCT, and Hb at the same time points between the groups (Table 4).

**Table 4:** Basic vital signs.

	Abdominal surgery group (*n* = 21)	Laparoscopic surgery group (*n* = 21)	Breast cancer surgery group (*n* = 21)	*P*-value
MAP (mmHg)				
Preoperative	102 (94, 105)	90 (81, 103)	93 (79, 100)	0.0811
After anesthesia induction	85 (75, 94) *	79 (71, 98) *	87 (77, 93)	0.7805
30 min after surgery	90 (81, 97) *	83 (80, 88) *	86 (80, 94)	0.3236
60 min after surgery	84 (79, 89) *	84 (79, 93)	85 (73, 91) *	0.9118
HCT (%)				
Preoperative	40 (37, 43)	39 (35, 42)	38 (35, 41)	0.3982
After anesthesia induction	37 (35, 39) *	36 (33, 39) *	35 (31, 38) *	0.4836
30 min after surgery	37 (34, 39) *	37 (30, 40) *	33 (30, 37) *	0.0523
60 min after surgery	36 (34, 38) *^#^	35 (30, 38) *	32 (30, 36) *^#^	0.0473
Hb (g/dL)				
Preoperative	12.8 (11.8, 13.6)	12.5 (10.6, 13.4)	12.9 (11.0, 13.4)	0.833
After anesthesia induction	11.6 (10.5, 12.5) *	12 (10.1, 13.0) *	12 (10.3, 12.7) *	0.9116
30 min after surgery	11.9 (10.7, 12.7) *	12.1 (9.2, 13.3) *	11.6 (9.8, 12.1) *	0.5012
60 min after surgery	11.4 (10.6, 12.7) *	11.8 (9.3, 12.9) *	11.1 (9.5, 11.9) *	0.4081

*P*-value derived from the one-way ANOVA or Kruskal–Wallis *H* test for continuous variables. The symbol * indicates a statistically significant difference between different times in the same group. The symbol # indicates that the difference between the two groups was statistically significant in the multiple comparative analysis.

The correlation analysis of SCr with FINS, C-peptide, and HOMA-IR showed that there was statistical significance (*p* < 0.05). The time-dependent parameter covariates (the coefficient value) of FINS and C-peptide were negative, and FINS and C-peptide had negative effects on SCr. The coefficient value of HOMA-IR was positive, and it had a positive effect on SCr. The correlation analysis of Cys C with FINS, C-peptide, HOMA-IR, and HOMA-β showed that there was statistical significance (*p* < 0.05). The coefficient values of FINS, C-peptide, HOMA-IR, and HOMA-β were positive, and they had positive effects on Cys C. The correlation analysis of β_2_-MG with FINS, C-peptide, and HOMA-IR was statistically significant (*p* < 0.05). The coefficient values were positive, and FINS, C-peptide, and HOMA-IR had positive effects on β_2_-MG (Table 5).

**Table 5:** Correlation analysis between the islet and renal function during surgery.

	*F* value	*P*-value	Coefficient value
SCr			
FPG (mmol/L)	0.29	0.5883	−0.0379
FINS (μIU/ml)	5.95	0.0160*	−0.1159
C-peptide (ng/ml)	16.71	<0.0001*	−0.01334
HOMA-IR	889.08	<0.0001*	0.05288
HOMA-β	1.00	0.3202	0.7983
HOMA-IS	0.42	0.5175	0.000296
Cys C			
FPG (mmol/L)	1.12	0.2912	−0.2736
FINS (μIU/ml)	12.24	0.0006 *	16.2850
C-peptide (ng/ml)	54.42	<0.0001*	1.6169
HOMA-IR	654.15	<0.0001*	4.1706
HOMA-β	5.88	0.0166 *	147.49
HOMA-IS	0.80	0.3715	−0.06641
β_2_-MG			
FPG (mmol/L)	2.08	0.1519	−0.2829
FINS (μIU/ml)	5.43	0.0212 *	4.5835
C-peptide (ng/ml)	37.25	<0.0001 *	0.3841
HOMA-IR	440.75	<0.0001 *	1.5909
HOMA-β	0.64	0.4235	50.9252
HOMA-IS	0.35	0.5562	−0.01181

The MIXED procedure of SAS was used to estimate the mixed linear model fitting for repeated-measures data with time-dependent covariates. A two-sided alpha value of 0.05 was used for all of the statistical tests. FPG: fasting plasma glucose; FINS: fasting insulin; HOMA-IR: homeostatic model assessment of insulin resistance; HOMA-β: homeostatic model assessment of β-cell function; HOMA-IS: homeostatic model assessment of insulin sensitivity; SCr: serum creatinine; Cys C: cystatin C; β_2_-MG: beta-2-microglobulin. A correlation analysis shows *p < 0.05.

The correlation analysis of FPG with HCT and Hb showed that there was statistical significance (*p* < 0.001). The coefficient values were negative, and HCT and Hb had negative effects on blood glucose. The correlation analysis between C-peptide and MAP showed that there was statistical significance (*p* < 0.05). The coefficient value was positive, and MAP had a positive effect on C-peptide. The correlation analysis of SCr with HCT and Hb showed that there was statistical significance (*p* < 0.05). The coefficient values were positive, and HCT and Hb had positive effects on SCr (Table 6).

**Table 6:** Correlation analysis of islet and renal function with MAP, HCT, and Hb.

	MAP			HCT			Hb		
	*T*-value	Coefficient value	*P*-value	*T*-value	Coefficient value	*P*-value	*T*-value	Coefficient value	*P*-value
FPG (mmol/L)	0.28	0.002724	0.7811	−5.32	−0.1498	<0.0001	−3.95	−0.5076	0.0001 *
FINS (μIU/ml)	1.39	0.08754	0.1682	−1.19	−0.4750	0.2377	−0.28	−0.4106	0.7830
C-peptide (ng/ml)	2.62	0.01497	0.0096*	−0.85	−0.03522	0.3952	−0.50	−0.07899	0.6198
HOMA-IR	1.03	0.001822	0.3116	3.80	−0.03472	0.0534	2.41	−0.1395	0.1230
HOMA-β	0.000	−0.5754	0.9653	3.59	−0.1052	0.0602	1.90	−2.0173	0.1705
HOMA-IS	0.59	0.000109	0.4429	0.25	−0.00194	0.6159	0.04	−0.00498	0.8382
SCr (mmol/L)	−0.97	−0.06681	0.3321	2.47	0.7689	0.0148 *	2.36	2.8661	0.0198 *
Cys C (μIU/ml)	−0.27	−0.00029	0.7843	1.17	0.008446	0.2436	1.48	0.03613	0.1423
β_2_-MG (ng/ml)	−0.36	−0.00112	0.7171	0.68	0.01259	0.4957	0.83	0.05571	0.4105

The MIXED procedure of SAS was used to estimate the mixed linear model fitting for repeated-measures data with time-dependent covariates. A two-sided alpha value of 0.05 was used for all of the statistical tests. FPG, fasting plasma glucose; FINS, fasting insulin; HOMA-IR, homeostatic model assessment of insulin resistance; HOMA-β, homeostatic model assessment of β-cell function; HOMA-IS: homeostatic model assessment of insulin sensitivity; SCr, serum creatinine; Cys C, cystatin C; β_2_-MG, beta-2-microglobulin. Considering the influence of time and group, two groups of variables are correlated (*p < 0.05).

We observed the incidence of intraoperative outliers of FPG, FINS, C-peptide, β_2_-MG, SCr, and Cys C. The incidence of intraoperative outliers of FPS in the breast cancer surgery group was 0, and there was a significant difference between the other two groups (*p* < 0.05). The incidence of intraoperative SCr outliers was higher in the laparoscopic surgery group and the breast cancer surgery group, reaching 61.90 and 75.00%, respectively, but there was no statistical significance compared with the abdominal surgery group (Table 7).

**Table 7:** Comparison of the proportion of islets and renal dysfunction.

	Abdominal surgery group (*n* = 21)	Laparoscopic surgery group (*n* = 21)	Breast cancer surgery group (*n* = 21)	*P*-value
FPG (mmol/L)	5 (23.81)	7 (33.33)	0 (0)	0.021
FINS (μIU/ml)	8 (38.10)	5 (23.81)	2 (10.00)	0.110
C-peptide (ng/ml)	7 (33.33)	8 (38.1)	4 (20.00)	0.430
HOMA-IR	7 (33.33)	7 (33.33)	5 (25.00)	0.801
SCr (mmol/L)	9 (42.86)	13 (61.90)	15 (75.00)	0.107
Cys C (μIU/ml)	4 (19.05)	2 (9.52)	6 (30.00)	0.252
β_2_-MG (ng/ml)	2 (9.52)	2 (9.53)	1 (5.00)	0.829

*P*-values were derived from the chi-square test for categorical variables.

## 4 Discussion

Endogenous hormone secretion, the inflammatory response, and subsequent hyperglycemia are changed due to anesthesia and surgery (such as increases in catecholamines, cortisol, growth hormone, and glucagon) (11). This prospective cohort study shows that there was no significant difference but rather a close correlation between the effects of different surgical methods on islet and renal function in patients who underwent non-cardiac surgery. This conclusion may be related to the limitations of this study. The sample size was small, and the observation time point was limited to 60 min. During the operation, FPG, FINS, and C-peptide gradually decreased after anesthetic induction but gradually increased 30 and 60 min after surgery. At present, there are very few studies on whether changes in islet function affect renal function. Our study found that after anesthetic induction, SCr, Cys C, and β_2_-MG initially decreased and then gradually increased after surgery. The effects of FINS, C-peptide, and HOMA-IR on Cys C and β_2_-MG were positive, and HOMA-β had a positive effect on Cys C. The changes in C-peptide had a negative effect on SCr, and there was a significant correlation between islet and renal function.

The islets are made up of five different types of cells: alpha (produces glucagon), beta (produces insulin), delta (produces somatostatin), PP (produces the pancreatic polypeptide), and epsilon (produces ghrelin) (12). FBG, FINS, and C-peptide are indexes reflecting the glucose metabolism function of islets. Reportedly, 20–40% of patients undergoing general surgery (13, 14) and approximately 80% of patients after cardiac surgery will have perioperative hyperglycemia. However, 12–30% of patients with intraoperative and/or postoperative hyperglycemia had no history of diabetes prior to surgery (13), which was described as “stressful high blood sugar” (11). Stress hyperglycemia usually subsides as acute disease symptoms or surgical stress are reduced. Perioperative hyperglycemia will increase the risk of postoperative infection (15). Studies have shown that patients with perioperative blood glucose exceeding 7.97 mmol L^−1^ have a higher risk of death (16). It is generally believed that only insulin resistance, without β-cell dysfunction, will lead to disturbances in glucose metabolism or diabetes. Impaired β-cell function is a necessary condition for the development of diabetes mellitus. Therefore, any increase in blood sugar means that β cell function is impaired. Surgical stress metabolic disorders are related to the degree of surgical trauma; the more serious the trauma, the more obvious the reactive hyperglycemia (17–19). Controlling blood glucose levels within the normal range during surgery has been shown to improve prognosis (20, 21). Our results in this study show that FPG declines after anesthetic induction, gradually increases after the start of surgery, and exceeds the preoperative level within 1 h. Although FPG was not correlated with MAP, it was negatively correlated with changes in HCT and Hb, which was consistent with the results of previous studies (22, 23). The incidence of FPG outliers in abdominal surgery was higher than that in body surface surgery, which further verified the effect of surgical stress on blood sugar.

Under normal conditions, blood glucose is an important factor that stimulates insulin secretion. However, when islet β cell dysfunction occurs, the curves are separate (3, 24). Consistent with our findings, blood glucose and insulin had the same trend of intraoperative changes, which gradually increased with increasing trauma after the start of the operation; intraoperative glucose levels and glucose–insulin-based metrics do not correlate well with insulin resistance (25). Previous studies have shown that insulin resistance may be an important mechanism in the development of postoperative complications (26). Therefore, we applied the homeostasis model (Homa Model) method in this experiment to further evaluate islet function.

The C-peptide is a negatively charged polypeptide composed of 31 amino acid residues and does not have a tertiary structure under physiological conditions (27). The C-peptide test is considered a powerful method to evaluate the function of pancreatic islets. The C-peptide levels decreased after the induction of anesthesia in patients in the abdominal and laparoscopic surgery groups in this study and gradually increased after the start of surgery. Consistent with the trend of insulin change, patients in the breast cancer group had a persistent elevation in C-peptide. The change in C-peptide was positively correlated with the MAP, and the incidence of abnormal values in the abdominal and laparoscopic surgery groups was significantly higher than that in the breast cancer group, both of which were lower than the normal value. Additionally, islet β cell secretion decreased. It has been further proven that abdominal surgery is more traumatic and that the stress reaction is higher than that of breast cancer surgery.

Hyperglycemia and insulin resistance have been recognized as causes of complications in cardiac surgery (28), surgical ICU (19, 20), and major abdominal surgery (29). The simpler HOMA method (including HOMA-IR, HOMA-β, and HOMA-IS) has been used as an alternative to the hyperinsulinemic normoglycemic clamp in studies of surgery-induced insulin resistance (30). Insulin resistance transiently occurs as a fundamental reaction to injury, including trauma and surgery, but also in response to other types of physical stress, such as fasting (30), pain (31), and immobilization (32). Previous studies reported a 7-fold preoperative change in m-values (a measure of insulin sensitivity) in non-diabetic surgical patients (32). Insulin resistance is closely related to complications in elective surgery (25, 28). This is consistent with our results. HOMA-IR gradually increased with the extension of the operation time.

The HOMA-β index in normal individuals was 100%, which was used to evaluate the individual indicators of islet β cell function (33–35). The better the β cell function, the better the insulin sensitivity, and the lower the blood glucose level. This is consistent with our study; that is, blood glucose levels were negatively correlated with β cell insulin secretion function and insulin sensitivity.

The occurrence of AKI is not uncommon in the perioperative period and remains an underestimated but clinically important complication (36). Even small elevations in creatinine have been associated with increased perioperative mortality and longer lengths of stay (37–39, 40, 41). Therefore, predicting the risk factors and preventing diseases for postoperative AKI are important issues for perioperative patient management (38). In cardiac surgery, a mere 20 mg/dL increase in the intraoperative blood glucose concentration is associated with an approximately 30% increase in pulmonary and renal complications and death (42). The results of our study showed that islet and renal function were highly correlated. FINS and C-peptide had negative effects on SCr and had positive effects on Cys C and β_2_-MG. HOMA-IR was positively correlated with renal function, and HOMA-β was positively correlated with SCr but did not show a correlation with FPG or a correlation with FPG and HOMA-IS. Our study found that compared to that of the patients in the laparoscopic surgery group, the SCr of the patients in the abdominal surgery group continued to increase and was comparable to the preoperative level 60 min after the start of surgery, whereas the SCr of the patients in the breast cancer group continued to decrease and was lower than that before surgery, indicating that abdominal surgeries have a higher impact on renal function than body surface surgeries. Similar to previous findings, cardiac surgery with CPB had the highest risk of AKI, followed by general surgery, thoracic surgery, orthopedics, vascular surgery, and urologic surgery (43). In addition, the changes in HCT and Hb had a positive effect on SCr, indicating that the occurrence of AKI was closely related to intraoperative hemorrhage (44).

The current clinical diagnostic indicators that reflect early functional damage to the kidney are SCr, blood urea nitrogen (BUN), and creatinine clearance (Ccr). Cys C is a cysteine protease inhibitor produced by all nucleated cells in the body, and it is released at a constant rate (45), is almost completely reabsorbed, and is degraded by the proximal convoluted tubule after passage through the glomerular filtration membrane (46). Therefore, the Cys C concentration in the serum is a very sensitive endogenous marker reflecting glomerular filtration function (47). Similar to Cys C, β_2_-MG is also a small molecule protein that can freely pass through the glomerular filtration membrane, and approximately 99.9% is reabsorbed and broken down in the proximal tubule. Therefore, β_2_-MG is elevated when glomerular filtration function declines. β_2_-MG can be used as an ideal indicator to evaluate the function of glomerular filtration (48).

Because of its constant rate of generation, the plasma Cys C concentration may be a better marker of the GFR than serum creatinine, and it has recently been proposed to replace serum creatinine in the routine assessment of the GFR rather than as a biomarker of AKI (49, 50). The results of our study showed that Cys C in patients in the abdominal surgery group was higher than the preoperative level, whereas Cys C in patients in the laparoscopic surgery group was lower than the preoperative level; however, the levels in the breast cancer group were within the normal range, indicating that there was no early renal function impairment. Cys C had no correlation with the MAP, HCT, or Hb and was not affected by hemodynamic fluctuations or an increased bleeding volume. The effect of the alterations in FINS, C-peptide, HOMA-IR, and HOMA-β on Cys C was positive.

Serum β_2_-MG is an indicator that reflects glomerular filtration function, and elevations in β_2_-MG levels in the blood are more sensitive than serum creatinine. Multiple studies have found that the β_2_-MG level is an early predictor of AKI in critically ill children (51), renal transplant patients (52), and liver transplant patients (53, 54). An additional study found that the concentration of serum β_2_-MG increased with every 100 μg/L, thereby increasing the prevalence of AKI by 6.4% (55). The results of this study showed that β_2_-MG decreased after the induction of anesthesia and gradually increased 30 min after surgery. Sixty minutes after surgery, β_2_-MG continued to increase in patients in the laparoscopic surgery group and exceeded the preoperative level, while in patients in the abdominal surgery group and the breast cancer group, β_2_-MG continued to decrease and was lower than that before surgery. The incidence of abnormal values of β_2_-MG at each time point was low, and it was not affected by surgical stress or increased blood loss. However, the changes in FINS, C-peptide, and HOMA-IR have a positive effect on β_2_-MG.

Compared with cardiac surgery, there are fewer studies on the occurrence of acute kidney injury in non-cardiac and non-vascular surgery, possibly because of its lower overall incidence. At present, related studies on islet and renal function have mainly focused on diabetic nephropathy (56) and the preoperative (57) and postoperative effects of hyperglycemia on renal function (2, 58, 59). This is the first study to explore the influence of different surgical methods on intraoperative islet and renal function. In this study, we were surprised to find that both the gold standard SCr of renal dysfunction and the latest biomarkers, Cys C and β_2_-MG, suggest a close correlation between islet and renal function. The influence of FINS and C-peptide on SCr is negative, and the influence of HOMA-IR on SCr is positive. This shows that SCr is not sensitive enough to detect early kidney injury, and only after the islet function is impaired can it show a positive effect. Changes in FINS, C-peptide, and HOMA-IR have positive effects on Cys C and β_2_-MG, indicating that Cys C and β_2_-MG can be used as ideal indicators for early kidney injury (59–62). It also proves that the design of this study is reasonable and has certain clinical significance.

We acknowledge several limitations of our study. The advantages of this study are the prospective design, detailed patient characteristics and short-term outcome data, low dropout rate, and accurate data. This is the first study to observe the changes in pancreatic islets and renal function during surgery at the same time, and there are few studies on the correlation between the two major organs. The limitations include the relatively small sample size. Due to the limitation of the length of the operation, we had fewer observation time points and did not thoroughly evaluate the incidence of postoperative acute kidney injury and the follow-up postoperative mortality. This study is a preliminary study of the effects of different surgical methods on islet and renal function. The connection between the pancreas and the kidneys has been proven in patients with diabetes, with RAS being involved in expression. Studies on the related mechanisms still need to be carried out to explore whether the two organs are related through the RAS.

## 5 Conclusion

Surgical stress induces intraoperative changes in pancreatic islet function in patients undergoing non-cardiac surgery. FINS, C-peptide, and HOMA-IR were positively correlated with Cys C and β_2_-MG. However, FINS and C-peptide were negatively correlated with SCr, HOMA-IR was positively correlated with SCr, and pancreatic islet function was significantly correlated with early renal injury. Whether this correlation would lead to later organ damage needs to be confirmed by further studies.

## Data availability statement

The original contributions presented in the study are included in the article/supplementary material, further inquiries can be directed to the corresponding authors.

## Ethics statement

The studies involving humans were approved by the Ethics Committee of Qianfoshan Hospital, Shandong Province. The studies were conducted in accordance with the local legislation and institutional requirements. The participants provided their written informed consent to participate in this study. Written informed consent was obtained from the individual(s) for the publication of any potentially identifiable images or data included in this article.

## Author contributions

YS and YW provided substantial contributions to the conception or design of the manuscript. XZ, MZ, YG, TS, ML, XG, YL, ZG, LC, and XD contributed to the acquisition, analysis, and interpretation of the data. YS, YW, XZ, MZ, YG, TS, ML, XG, YL, ZG, LC, and XD participated in drafting the manuscript, and YS revised it critically. All the authors have read and approved the final version of the manuscript.

## Figures and Tables

**Table:** 

FPG	Fasting plasma glucose
FINS	Fasting insulin
SCr	Serum creatinine
Cys C	Cystatin C
β_2_-MG	Beta-2-microglobulin
HOMA-IR	Homeostatic model assessment of insulin resistance
HOMA-β	Homeostatic model assessment of β-cell function
HOMA-IS	Homeostatic model assessment of insulin sensitivity
CKD	Chronic kidney disease
AKI	Acute kidney injury
DN	Diabetic nephropathy
BIS	Bifrequency index
MAC	Minimal alveolar concentration
FiO_2_	Oxygen concentration
BMI	Body mass index
GLB	Globulin
TP	Total protein
ALB	Albumin
ERAS	Enhanced recovery after surgery
RAS	Renin-angiotensin system
EGP	Endogenous glucose production
